# Towards More Sustainable Diets—Attitudes, Opportunities and Barriers to Fostering Pulse Consumption in Polish Cities

**DOI:** 10.3390/nu12061589

**Published:** 2020-05-29

**Authors:** Agata Szczebyło, Krystyna Rejman, Ewa Halicka, Wacław Laskowski

**Affiliations:** Institute of Human Nutrition Sciences, Warsaw University of Life Sciences WULS-SGGW, Nowoursynowska 159c, 02-776 Warsaw, Poland; krystyna_rejman@sggw.edu.pl (K.R.); ewa_halicka@sggw.edu.pl (E.H.); waclaw_laskowski@sggw.edu.pl (W.L.)

**Keywords:** pulses, beans, plant protein, consumption, sustainable diets, Poland, cities, employees

## Abstract

Despite the evidence-based health benefits of pulses and their significant role in sustainable diets, consumption remains at a very low level in highly developed countries. In an attempt to fill in the knowledge gaps on factors influencing this phenomenon, a study aimed at identifying attitudes, incentives and barriers to pulse consumption was carried out in a sample of 1027 Polish urban employees aged 25–40 years. The sample (quota type) was representative in terms of age and gender. Exploratory classifications using Kohonen neural networks were performed to define profiles of participants for each analysed issue. Pearson’s chi-square analysis was used to check whether the profiles depended on socio-demographic characteristics of the respondents. The results suggest that very low pulse consumption is a result of lack of habits, discomfort after eating and long preparation time. Pulses were recognized as a good source of protein (72% of the sample), especially among women (81%). Only 43% of the sample saw pulses as a substitute for meat. The majority of consumers pictured pulses as a tasty and healthy food, although they were not sure if this is true for small children. Women recognised pulses as a more environmentally friendly food but this knowledge would not impact their intake. Profiles of respondents with positive attitudes towards increased pulse consumption were identified, constituting 39% of the sample. These consumers could eat more if they were encouraged to do so. This shows that programmes aimed at fostering greater pulse consumption are crucial to activate a change towards more sustainable diets. At the same time, simple and clear guidelines should be developed to overcome the unjustified stereotypes about pulses. These would support consumers to make healthier and more sustainable choices and help professionals carry out effective promotion and education activities.

## 1. Introduction

Pulses, one of world’s traditional staple food crops, are a subgroup of legumes harvested for dry edible seeds. Among them, the most important in human nutrition and trade are beans, lentils, peas, and chickpeas. The category does not include green peas and fresh beans which are systematised as vegetables, nor oil legumes (soybeans and peanuts), as the classification adopted by the Food and Agriculture Organization is based on the world’s main ways of utilization [1]. In Polish, this definition of pulses is subject to limitations, as no separate term for legume seeds grown for dry seeds and those species of the *Fabaceae* family that have a higher fat content is used. The most prevalent classification concerns species used for food or feed. It is common for the group of dry edible legume seeds to include beans, peas, soybeans in recent years as well as lentils and chickpeas, and sometimes broad beans, although the latter are consumed mainly in the summer as a fresh vegetables [2]. 

The effects of pulses on human health can be related to the provision of energy and nutrients and to other phytochemical constituents that may have beneficial effects either on physiological function, e.g., gut health, or prevent various diseases, e.g., the risk of chronic disease [3]. Overall, this group of foods has high nutritional value and is relatively low in fat. Pulses are an abundant source of protein (20%–35%, on average) and complex carbohydrates (approximately 60%). They also provide vitamins (especially thiamin, niacin, folate, riboflavin, and pyridoxine) and an array of minerals such as iron, zinc, potassium, calcium, magnesium and selenium [4,5,6,7]. 

As a source of protein pulses are especially rich in the amino acid lysine, which is often low in cereal grains. Plant-based diets that include pulses can therefore provide the necessary set of amino acids in everyday nutrition [8,9]. Despite the fact that their high carbohydrate content pulses are slowly digested, placing them lower on the glycemic index (GI) scale than other carbohydrate-rich foods such as rice, white bread, or potatoes [10]. Due to their low GI, pulses are recommended for people with type II diabetes as they support glycemic control [11,12,13]. Pulse consumption is also emphasized in efforts to close the dietary fibre gap since they provide two to three times more dietary fibre per 100 g edible portion than whole grain cereal products, the type of foods frequently advertised as source of dietary fibre [14]. Moreover pulses contain a high level of resistant starch, which feeds gut bacteria responsible for intestine–brain communication and impacts the general health condition [15,16,17].

There is growing evidence of the role of pulses in the treatment of obesity, due to the high fibre content and control of satiety [18,19] as well as cardiovascular diseases [20]. Pulses have a unique nutritional profile consistent with several dietary composition factors thought to assist with weight control [21]. The phytochemicals, saponins, and tannins found in pulses possess antioxidant and anti-carcinogenic effects, indicating that pulses may have significant anti-cancer effects [22]. Pulses also contain several anti-nutritional substances (e.g., trypsin and chymotrypsin inhibitors, lectins, glycosides, phytates, and tannins) that can cause bloating and gastrointestinal discomfort, but these inconveniences can be eliminated by simple cooking techniques [23,24,25]. 

In many cultures, pulses are eaten daily in large amounts and are both affordable and accessible to the population [26]. In developed countries, pulse consumption has been displaced with animal products which have become the main source of protein [14]. Dietary patterns in the United States, Canada and European countries are characterised by low consumption of pulses and other plant food and high proportion of animal origin food, especially red and processed meat and other highly processed products. The observed nutritional transformation is one of the substantial drivers of The Global Syndemic (including obesity, undernutrition, and climate change) which threaten human health and survival, and contributes to the detrimental effects of food systems on the natural environment [27]. 

Guidelines to increase the consumption of pulses are justified by nutritional, but also socioeconomic and environmental reasons. The cultivation of these crops is many times cheaper than meat production, especially regarding water and soil efficiency and helps reduce greenhouse gas emissions [28,29]. The plants have nitrogen-fixing ability, which can improve soil fertility by catalysing the creation of high-quality organic matter in soils, resulting in increased farmland productivity. The cultivation of legumes promotes biodiversity and creates a more diverse landscape for animals and insects, and decreases the risks that farmers face from climate and market fluctuations [30,31,32]. Pulses are also of strategic importance in feeding the world, as economic benefits translate into more sustainable agricultural systems and higher food security [31,33].

Increasing the consumption of pulses constitutes an important component of the dietary shift towards more sustainable and healthy diets. The aim of the present paper was to investigate the attitudes towards pulses among Polish consumers and to understand the perceived opportunities and barriers to increasing consumption of these foods. Consumers aged 25–40 working in cities were selected for the study due to the long-term nature of the effects of the needed change in their consumption patterns. Increased pulse consumption would improve their nutritional and health status and help develop sustainable eating habits among their children. It would also protect the natural environment of the world in which they live and future generations grow up in. 

## 2. Materials and Methods

### 2.1. Data Collection and Sample

The survey data was collected using the Computer Assisted Web Interview (CAWI) method in November 2018. The questionnaire was distributed online among members of a consumer panel of 65,000 active participants by a commercial market research company. During the survey’s preparation process we cooperated with the research company to adapt the formula of questions to the panel service. Invitations with a link to the survey were sent (emailed) to individuals from the panel. Two criteria for participation in the survey were established: age between 25–40 years and “working in the city”. As the criterion of “working in the city” was imposed, the definition of work was adopted as the provision of continuous work based on an employment contract, or in the form of a civil law contract, or a self-employed business activity. The conducted pilot study involved 50 people. An estimated time of the completion of questionnaire was 15–16 min. Only when the questionnaire was properly filled in, the research agency directed remuneration to the respondent’s panel account. There were 1833 individuals who opened the survey link, 628 did not complete the questionnaire, 178 did not meet the criteria. Ultimately, the survey involved 1027 people (thus 56% of those who joined the survey), aged 25–40 working in cities located in all parts (16 voivodships) of Poland. The sample was of controlled quota type and was representative in terms of age and gender. It was based on data from the national statistical office Statistics Poland (GUS). The survey was approved by the WULS-SGGW Ethics Committee of Scientific Research with the Participation of People (approval code 13p/2018).

### 2.2. Survey Measures

This article focuses on survey data obtained from four selected sections of a broader research related to consumer behaviours towards pulses. It concentrates on the frequency of consumption, attitudes and barriers to consumption and chances for increasing pulse intake. These issues were investigated with the use of six complex survey questions, two of which regarded consumer attitudes, and two frequency. The respondents were asked to mark their answers on a 4-point scale, a 5-point Likert-type scale ranging from ‘definitely no’ to ‘definitely yes’, a multiple-choice box or a non-gradual scale with marginal values. One question implied free text open answers. A form of this part of the questionnaire is attached in the Appendix A.

It must be clarified that although the study focused on pulses, the respondents were also asked about their consumption of other legume-based products—specifically selected soy foods, i.e., tofu and other soy products and soymilk. Soybeans have a much higher fat content than pulses, however they are an adequate source of protein, iron, calcium and play a significant role in healthy diets [34]. Moreover, soy products can be important when analysing food consumption in the context of sustainability as they are included in the planetary healthy reference diet [35]. In Poland, the term ‘pulses’ traditionally refers to beans and peas, secondly to lentils, chickpeas and lastly to soy products which were widely introduced to the market in the last decade. Our research showed that the consumption of the latter foods remains on a very low level in the sample what lead to the decision to use the term ‘pulses’ in the paper in reference to the studied group of products. 

In an additional question, the respondents’ knowledge of the concept of sustainable consumption was examined. The studied consumers were firstly asked if they are familiar with the term (possible answers were ‘yes’ and ‘no’) and if the answer was ‘yes’, they had to select the definition of sustainable consumption. The following four answers were posted, out of which three were not correct:

- The energy value of food consumed equals the energy expended by the body
- In the food consumed there is the same share of plant and animal products
- Everyday diet is carried out so as to minimize the influence on the natural environment
- The cost of nutrition is adapted to the financial capabilities of the household.

In the correct (third) answer the definition of sustainable food consumption had been deliberately simplified to the environmental dimension as it was done in questionnaires in our previous research conducted in Poland [36]. 

Sociodemographic characteristics included each respondent’s year of birth, gender, size of place of residence, level of education, type of employment (full time or other) and labour type classification (blue collar etc.) Survey participants were also asked about their household’s size, if they had any children and to assess the financial situation of their household. Participants had an opportunity to indicate if they follow any special diet and how much of their food was usually prepared at home (intensity of food preparation). Moreover, the interviewees’ BMI scores were calculated (based on self-reported weight and height), although sharing this information was not obligatory. 

### 2.3. Data Analysis

Exploratory classifications using Kohonen neural networks available in the Statistica program were performed for four sets of survey questions presented in this paper (regarding consumption frequency, attitudes, barriers and opportunities for growth). It was assumed that searching for multidimensional classifications using these networks would explain the majority of variability. The classifications with average correlation ratio reaching or exceeding 0.5 were accepted for presentation in this paper. The averaged responses of a given group were called ‘the response profiles’ and presented on charts. Each chart shows all the profiles of the analysed set of statements and all its aspects. 

For each of the profiles, Pearson’s chi-square analysis (the significance level *p* < 0.05) was performed to test for association with gender and whether or not the household had children under the age of 18. Only significant associations are reported in the paper. A two-tailed z-test (*p* < 0.05) was performed to disaggregate the statements regarding the respondents’ opinions about pulse consumption in order to investigate the differences between men and women.

## 3. Results

### 3.1. Sample Characteristics

The average age of the study participants was 32 (±7) years and women constituted 52%. Table 1 shows the socio-demographic and household characteristics of the 1027 participants who completed the questionnaire. 

Almost half (48%) of the sample lived in cities with less than 100,000 habitants (31% in cities with less than 50,000), and another 23% lived in the five big Polish cities with 500,000+ habitants. The survey participants were well educated as 69% of them held a Bachelor (or higher) degree. There were three people on average in the respondents’ households and 51% of respondents had children under the age of 18. Almost half (48%) of the sample evaluated their household financial situation as good or very good and 48% as average. A vast majority (85%) declared full-time employment. Most of the participants admitted that they do not follow any specific diet (76%), 16% indicated that they limit their intake of animal products, only 3% considered themselves vegetarian (no meat), and 1% as vegan (no animal products in diet). Other dietary limitations concerned 4% of the respondents. 

Based on the calculated Body Mass Index, four groups of respondents were identified: underweight 10%; normal weight 45%; overweight 25%; obese 10% (the remaining 10% did not provide data). More than half of the respondents (56%) declared that they eat most of their meals at home and prepare them from scratch using basic, unprocessed food products. Almost a quarter (24%) estimated that they eat circa half of their food outside their homes. Eating mostly at home using convenience food for meal preparation was indicated by 15% of respondents. Only 5% of the group declared that they eat mostly outside of their home.

Interestingly, 39% of respondents declared that they were aware of the concept of sustainable consumption. Among them, 43% pointed at the correct definition indicating the relationship between food and the state of the natural environment. Therefore, 17% of the whole sample correctly defined sustainable consumption. Such a result demonstrates a higher awareness and knowledge of the concept among Poles, compared to our previous studies. According to a survey carried out in the Mazovia region in 2014 only 6% of big city dwellers were familiar and correctly recognized the term [36]. In rural areas the percentage was slightly higher (9%) in 2017 [37]. 

### 3.2. Frequency of Pulse Consumption

The frequency of pulse consumption among the studied sample of Polish city employees was low, as on average it was a few times a year (mean 2.02). Beans were consumed most frequently (2.49) (Table 2).

**Figure 1:**
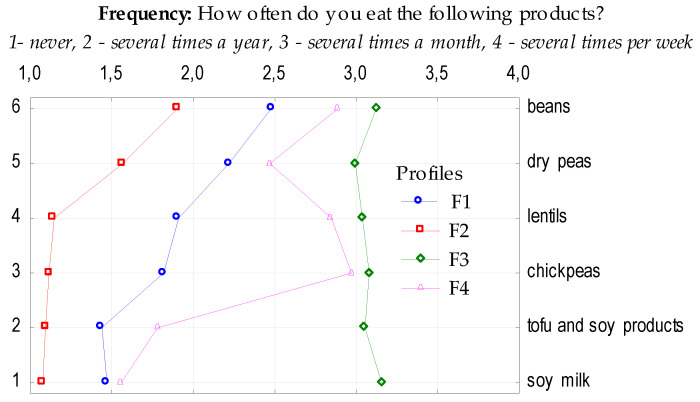
Profiles of respondents regarding frequency of pulse and soy product consumption.

People in the biggest profile (F1), which contributed to 35% of the studied group ate pulses several times a year (mean 1.89) (Figure 1). They preferred beans, secondly peas, lentils and chickpeas (mean 2.48–1.82) and almost never tofu or other soy products (mean 1.44–1.47). In the second profile (F2; 30%), pulses were consumed the least often (mean 1.32). People in this profile occasionally consumed beans (mean 1.90), they never consumed peas (mean 1.57), lentils (mean 1.15), chickpeas (mean 1.12) or soy products (mean 1.09–1.08). The third profile (F3), which constituted 18% of the total sample ate all kinds of pulses and soy products several times a month (mean 3.07), whereas soy milk, beans and chickpeas were consumed more frequently. The fourth profile (F4; 17%) declared also eating the total group of studied products several times a year, although chickpeas, beans and lentils intake was more frequent—several times a month (mean 2.97, 2.89, 2.85, respectively). 

**Figure 2:**
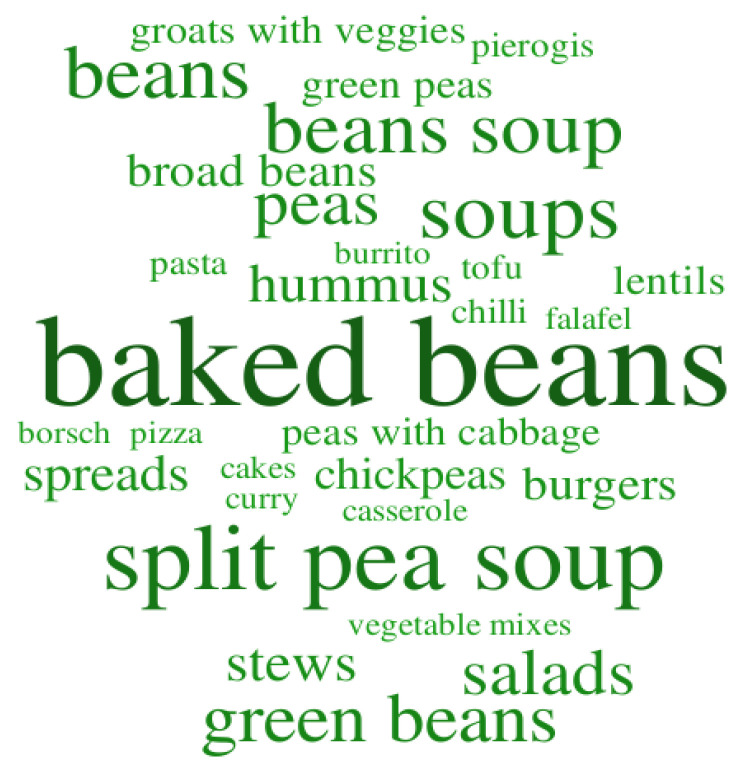
Word cloud based on most popular pulse dishes in the group of urban employees. (Word cloud provided by worditout.com under the Creative Commons License.)

Respondents were asked to name three dishes made from pulses that they had eaten recently. World cloud (the font size depends on the frequency of declarations) indicates that baked beans is a dish mentioned most frequently (32%) (Figure 2). Other traditional Polish dishes, such as split pea soup (20%) or bean soup (14%), were also popular. Respondents also recalled green beans (13%) and fresh broad beans (6%) which are widely consumed vegetables in the spring and summer seasons in Poland. Dishes like hummus (8%) and different bread spreads (7%) were also mentioned by the survey participants. Burrito, curry, falafel which are dishes sourced from other cultures’ cuisines, where represented on the list by only 1% of indications. 

### 3.3. Attitudes towards Pulse Consumption

**Figure 3:**
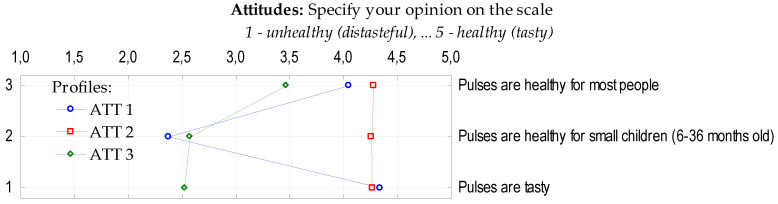
Profiles of respondents regarding attitudes towards health and taste of pulses.

The interviewed Poles claimed that pulses are rather healthy (mean 4.01) and rather tasty (mean 3.92). They were more doubtful about the health benefits for small children (aged 6–36 months) (mean 3.12). When analysing the identified three profiles related to the health benefits of pulses (Figure 3), respondents differed mostly in their attitudes towards the health benefits for small children. Respondents from the biggest profile (ATT1), which grouped 41% of the total sample (56% were females, 47% individuals had children), indicated that pulses were rather unhealthy for small children (mean 2.38), while rather healthy and tasty for most people (mean 4.05 and 4.34, respectively). Consumers in the second profile (ATT2; 37%) declared that pulses are tasty (mean 4.26) and healthy for most people (mean 4.28) as well as for small children (mean 4.26). This profile was structured by 54% of women and 60% of people with children under 18 years. Over one fifth (ATT3; 22%) of the studied consumers, among which 60% were male and 58% of participants had no children, presented an ambiguous attitude towards the health benefits of pulses for most people (mean 3.47). They were doubtful about the health benefits for small children (mean 2.57) and to the tastefulness of this food (mean 2.52).

The respondents in general expressed moderate or definite agreement with the 11 statements reflecting opinions and attitudes towards pulse consumption (Table 3). The majority (64%) of them agreed that ‘pulses (without meat) can be a base for a proper meal’. According to the results of the two-tailed z-test, significantly more women (72%) and significantly less men (54%) rated their opinions higher on the scale. Almost half (45%) of the sample did not agree with the statement that ‘dishes made of pulses are too common’ and 40% disagreed that ‘pulses are good only for people who do not eat meat’. Most of the participants (72%) acknowledged that ‘pulses are a good source of protein’ and only 6% disagreed with this statement. Significantly more women, compared to the total sample, perceived pulses as a good source of protein (81%), and significantly fewer men (62%). At the same time, almost one-fourth (24%) disagreed that ‘meat can be replaced by pulses in everyday cooking’ and 43% agreed with this statement, and again a more positive attitude was marked by significantly more women (52%) and less men (33%).

With regards to the environment, 62% of respondents agreed that ‘pulses are more environmentally friendly than meat’, 29% remained neutral, although respondents were more sceptical towards the idea that ‘substituting meat with pulses slows down climate change’ (41% neutral, 20% disagreed). 

### 3.4. Barriers

In the questionnaire part which focused on pulse consumption barriers, survey participants could indicate any number of the eight answers, which included ‘other’ and ‘no barriers’. They considered the ‘lack of eating habits’ as the main barrier to pulse consumption (30% of the sample), followed by the ‘feeling a discomfort after eating’ (29%). One fourth (25%) of individuals indicated ‘long preparation time’ as a factor limiting the consumption of pulses. Concern about ‘the lack of acceptance of dishes made from pulses by all household members’ was marked by 20% of consumers. ‘Lack of preparation skills’ and ‘non-acceptance of taste’ were chosen as barriers to pulse intake by 16% and 12% of respondents, respectively. ‘No barriers’ were declared by 17% of individuals. 

The conducted statistical analysis identified four profiles of consumers based on the perceived barriers. The largest of them (44%), of which 60% were men, indicated various barriers, mainly ‘long preparation time’ and ‘discomfort after consumption’. Respondents in this profile did not see ‘taste’ or ‘lack of habit’ as barriers to pulse consumption. The second profile (27%), consisting of 70% of women, predominantly chose ‘lack of habits and traditions’ as the main barrier. The third profile showed that people who answered that they ‘do not see any barriers to pulse consumption’ did not indicate any other factors (17% of a total sample, 56% women). Participants, who perceived the main barrier in lack of taste acceptance, structured the fourth profile (12% of the total sample), 63% of them men. 

### 3.5. Chances for Growth

The study participants as a whole sample rather agreed that new recipes (mean 3.86), gaining preparation skills (mean 3.74), lack of discomfort after eating (mean 3.68), lower prices of products offered on the market (mean 3.56), gaining the knowledge about pulse nutritional value (mean 3.55), wider offer in restaurants (mean 3.54) were factors that would increase pulse intake and reduce the proportion of meat and processed meat in their daily diets. A belief that through eating pulses they may protect the environment (mean 3.44), wider offer of ready-to-eat (RTE) products (mean 3.38) and educational campaigns (mean 3.38) were perceived as neutral drivers of change. Producers’ advertisements were seen as the least impactful measures (mean 3.17). 

**Figure 4:**
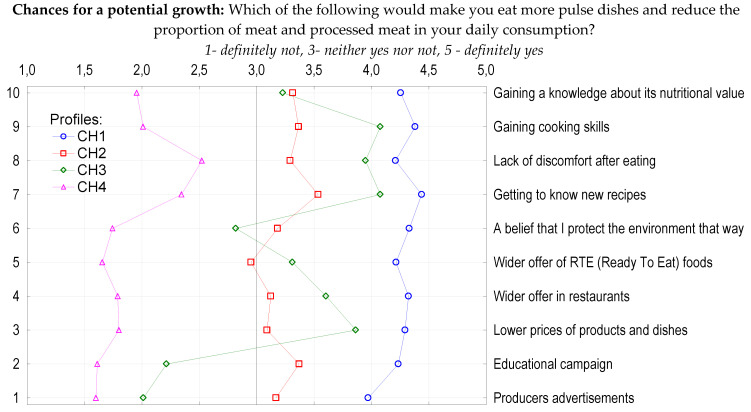
Profiles of respondents based on their statements about chances for pulse consumption. Assessment: 1.0–1.50—totally disagree; >1.50–2.50—rather disagree; >2.50–3.50—neutral; >3.50–4.50—rather agree; >4.50–5.00—totally agree.

Further analysis (Figure 4) showed that the highest number of respondents, 39% of the sample, was in the CH1 profile, representing the most positive attitude towards all the listed actions and indicating them as a chance for increasing pulse consumption (means 3.97–4.44). This group was comprised of 58% females and more than half (54%) of the profile members had children. The second profile (CH2; 37%) combined respondents who neither agreed nor disagreed that any of the specified options would change their usual pulse intake (means 2.95–3.37) with the exception of ‘learning new recipes’ which they rather agreed could increase their pulse consumption (mean 3.53). This profile had almost the same share of males and females (51% vs. 49%) and people who had and did not have children (52% and 48%, respectively). The third profile (CH3; 15%) rather agreed that the biggest chance for increasing consumption is ‘gaining preparation skills’ as well as familiarity with new recipes (equally mean 4.08). Gaining a belief that higher pulse consumption contributes to environmental protection was a neutral factor of change (mean 2.82). Consumers in this profile rather disagreed that an ‘educational campaign’ (mean 2.21) or ‘producers’ advertisements’ (mean 2.01) would convince them to consume more pulses. This profile was described by 53% men and 45% of people with no children. The fourth profile (CH4) gathered 9% of respondents who did not perceive the proposed factors as potential motivators for change (means 1.60–2.34). Even the highest scored option, ‘lack of discomfort after eating pulses’ (mean 2.52), was also seen as a neutral driver for eating more pulses. Most of the respondents in this profile were male (64%) and people without children (62%). 

## 4. Discussion

This study provided data about attitudes, incentives and barriers to pulse consumption among consumers aged 25–40 years and living and working in Polish cities. Other authors found that higher consumption of pulses was reported among younger, highly educated adults living in big cities [38,39,40,41]. 

The chosen statistical methods aimed at identifying consumer profiles in the surveyed group provided an insight into pulse consumption and presented differences in the attitudes of survey participants. It was observed that each of the pulse species and products, as well as whole group, may entail different consumer perception and result in different levels of intake. A study from South Italy showed that women were more frequent pulse eaters; however, in our sample gender did not contribute to significant differences between profiles [40]. Although differences were not very significant, we observed that lentils and chickpeas were more popular among younger consumers (25–34 years) and living in the biggest Polish cities. A French study also found that red lentils had a different position among other pulses and was described as more fancy and chosen more frequently by young consumers from the cities [42]. Whereas a study among Fins indicated that social image was a driver to change towards plant proteins, and it was mainly women who were ongoing that process [41]. In our study more than half of the individuals agreed that eating pulses has become trendy and it was significantly more women who agreed with that statement (62%). 

The very low pulse intake among our research participants is consistent with the statistical data gathered on population level. According to FAO Food Balance Sheet data (FBS 2019), the average consumption of pulses (estimated at supply level or availability of given food for human consumption) in 2017 in the EU was estimated at 2.75 kg per capita, compared to 7.57 kg per capita worldwide. In the case of Poland, FAO data show a rise from 1.86 kg in 2014 to 2.01 kg in 2017. The data from the Polish Household Budget Surveys carried out in a representative sample of the households by Statistics Poland office indicate that consumption of pulses at home amounted to 0.6–0.9 kg per year in the last two decades. The EAT Lancet Commission on healthy diets from sustainable food systems recommends dried beans, lentils and peas consumption to be at the average level of 18.25 kg/capita/year with the addition of over 9 kg of soy products and another 9 kg of peanuts [35]. Based on this data, there is a need for a 10-fold increase in the consumption of pulses in relation to food balance sheet data, or over 20-fold, taking into account data from household budget surveys in Poland. A minimum serving size of 100 g of cooked pulses per day was proposed by a Canadian research team [43] as a reasonable target for aligning strategies that promote the dietary and nutritional attributes of this food group. This amount can address the global nutrition challenges with regards to protein and energy malnutrition in developing countries, but also iron, folate, zinc and potassium insufficiency, and the too low fibre intake worldwide. The pulse intake recommendations contribute to the idea of implementing sustainable diets, which can be interpreted in many ways [44]. However, the central and most practical aspect always concern a switch to more plant-based dietary patterns, where protein intake of animal origin should be reduced to a minimum and the consumption of plant-based proteins, such as pulses, seeds and nuts should increase [45,46]. A growing body of evidence highlights benefits of a flexitarian diet which may be seen as a compromise and result of health, environmental, and animal welfare concerns [47]. EAT Lancet described a flexitarian diet as a planetary health diet which has become the main goal in changing the global food system [35]. This desired dietary pattern endorses that daily nutrition is based on a diversity of products of plant origin, low amounts of animal foods, mainly unsaturated rather than saturated fats, and small amounts of refined grains, highly processed foods, and added sugars. Such a diet has an appropriate caloric intake. In practice, the term flexitarian includes part-time meat consumers, who describe themselves as vegetarians occasionally eating meat as well as those who do not eat meat every day [35,48]. 

A knowledge of consumer attitudes towards pulses is key for starting the process of increasing their consumption and making diets more sustainable. Limiting the amounts of animal protein in daily nutrition is a driver to higher intake of plant proteins [49], but only 4% of our sample did not eat meat or animal products and 16% limited these products in their diets. The 2019 report on consumer attitudes towards plant-based products showed that 8.4% of adult Poles refrain from consuming meat or animal products on a daily basis [50]. According to the IPSOS report conducted in 28 countries, vegan and vegetarian diets have become more popular in the last years and are followed by 8% of these populations. In Europe, recent studies show significant differences between countries; for example, 10% of adults follow a meatless diet in the UK compared to 1.4% in Switzerland [51,52]. In 2019, an integrated and dynamic four-phase model of the conversion process to a more plant-based diet and reducing meat consumption was described by a Swiss research team [52]. The first phase was expressed as ‘I never considered reduction meat consumption’, the second ‘I’ve considered reducing my meat consumption, but I haven’t yet put this plan into practice’. In the third phase consumers claimed, ‘making sure that I consume less meat occasionally’, which corresponds with 16% of individuals from our study who declared that they limit meat and animal product consumption. The fourth phase was described as ‘I take consuming little or no meat for granted’. 

In the present study, knowledge about the nutrition benefits of pulses was considered as an incentive to increased consumption of this food. However, among French consumers that was not a big enough reason for a real change [42]. In another Finish study, environmental awareness was not convincing enough to make consumers eat more pulses [39]. On the other hand, natural concerns, health, and weight control motives were found as significant to those individuals whose diets included beans and soy products [41]. In our study, especially women presented more pro-environmental attitudes, declaring that pulses are more environmentally friendly than meat (70%) and pictured pulses as a meat replacement (52%). Those results are in line with findings that women had more positive attitudes towards a pro-environmental protein intake [53]. Similarly, a study among Polish big-city dwellers showed that environmental concerns play the biggest role among the ‘adopters’ for the sustainable diet cluster which was dominated by women [36]. Based on our data, the awareness about pulses being a source of protein seems not sufficient enough to make more of respondents think that these products can replace meat in everyday cooking. This can be related to the cultural understanding of pulses in Poland. Familiar and recognizable meal format in our part of Europe and other developed countries includes staples such as potatoes, vegetables and a protein component, which is typically meat. This might influence the willingness to replace meat with plant-based proteins [53]. Moreover, products such as peas, beans and potatoes are typically considered as low-income household foods, and during economic growth meat becomes a preferable product [54]. Additionally, more practical concerns are recognized from other studies from high income countries. Taste or disliking pulses was a main obstacle among Canadian [38] and French [42] consumers, as well as a lack of skills for preparation or difficulties cooking them. Inconvenience was also found as a barrier to replacing meat with plant protein in the study among Fins [41]. Our study is in line with those findings as, for one-fourth of the participants, long preparation time was a barrier to pulse consumption. The process of soaking and cooking pulses is time consuming and time availability and employment was a significant factor influencing home cooking according to a systematic review [55]. Gaining skills and new culinary recipes are potentially the most important drivers for increasing their consumption according to our findings, which support one of the IPSOS Report conclusions that cookbooks and recipe websites should be a crucial component of communication about pulses with consumers [38]. A study in Finland suggested that those individuals who know how to prepare beans indicated higher level of intake [39]. Similarly, the majority of Australian frequent pulse eaters indicated that they are familiar with cooking legumes [56]. A wider offer of pulse dishes in restaurants and cafeterias also would encourage consumers and transform pulses perception as well as make their taste more familiar [41]. In our sample consisting of young people working in the cities who predominantly eat and prepare most of their meals at home, 56% of consumers agreed that a wider offer in restaurants would be an impulse for higher pulse intake. A wider offer of products inspired by cuisines of different cultures helps introduce dishes traditionally made with pulses, which can lead to an increased awareness of European consumers on their extremely varied use [1,39]. Particularly, Mexican, Indian or Middle Eastern cuisines influence consumer perceptions of pulses [56]. Still, our research found that the ‘top of mind’ dishes made from pulses are those which are traditional to Polish cuisine and so they stay marginal or may be seen as too common. As a cultural contrast, results from a study conducted among Hispanic and non-Hispanic white women in the US are worth noting. A high level of bean consumption was observed, as the vast majority disagreed that it was difficult to make a meal with beans and at the same time did not care about the acceptance of this type of food by the family and children [57]. Conversely, the concern about acceptance of dishes made from pulses by all household members was observed among respondents in our study, especially women. Their doubts about the healthiness of pulses for small children may be an indicator of lack of knowledge about the nutritional value of pulses. Even among health professionals, knowledge gaps about the benefits of eating pulses were reported [58]. It can be assumed that those concerns are connected with a discomfort after eating which was established in our study as the second most important barrier to pulse consumption, and reported also in a study among Australians [59]. Meanwhile, simple culinary techniques are efficient in lowering side effects such as flatulence and it is crucial to promote this information [1,24,25]. Another aspect of including pulse dishes in sustainable healthy diets is to prepare them without a meat or animal fat component which is popular in Poland and in many cases may cause the discomfort. In addition, infrequent pulse intake, which is a significant source of fibre and resistant starch [14], may lead to excessive intestinal gas in those individuals who have an overall low fibre intake and can result in avoiding those dishes in the future. The body is able to adjust to a higher fibre intake when pulse consumption continues [60].

This paper provided the insight about behaviours of young Poles working in cities. In other study, this group was established as being potentially the most aware of the health and environmental benefits of pulse consumption and with the highest intake level of this food. However, our data showed that even among this specific population group pulses are consumed very infrequently. This insight may suggest that clear and straight communication about the benefits of this food group as well as recommended portion size and frequency of consumption is essential in creating relevant nutrition information. This finding can help health professionals, nutrition educators and dietitians to underline the importance of pulses in the daily eating habits of the population. Pulses have a double burden of their image. The first refers to them as being staple, common food, which moreover is perceived as not healthy for everybody. On the other hand, they can be treated as a fancy and rather expensive ‘superfood’ which brings them closer to a fad diet concept. 

Policy makers, NGOs and specialists can promote higher pulse consumption based on the findings of our study, for instance:

- Focus on women who have already a more positive attitude towards pulse consumption and encourage them to introduce pulses to the daily nutrition of their families (including small children);
- To inform both women and men about the reasons of flatulence after eating pulses and ways of eliminating it with focus on more frequent consumption as the most efficient solution to the problem;
- Underline the practical aspects of preparing and eating all kinds of pulses as a convenient way to prepare a nutritious meal;
- To promote the nutritional benefits of eating pulses among both women and men;
- To raise awareness about the role of pulses in increasing diet sustainability, which can foster consumption and enhance the message, especially among women.

The results from our study are helpful in establishing what actions or nudges would be most efficient in promoting pulse consumption. Pulses are most often classified as an optional source of protein, as in the case of Polish dietary guidelines [61], although American MyPlate introduced them in two food categories, proteins and vegetables [62]. It was also reported that sustainability aspects should be included into dietary guidelines [63]. The fact that the classification of pulses is not clear and somewhat neglected makes nutrition communication and consumer education regarding pulses challenging [64]. The unique nutrient composition, the significant role in a sustainable diet and concomitant lack of knowledge as well as low levels of intake suggest that clear recommendations for this food group, separated from others, would bring beneficial changes for both human health and the environment. 

### Strengths and Limitations

The main strength of this study is linked to the quota type sample which is more reliable than convenience methods. However, a study on a representative sample is recommended for further research as this study was not representative for the Polish population. Notably, this is one of the few studies that provides data about attitudes towards pulse consumption among European consumers. To the best of our knowledge this is the first study about pulse consumption among Poles. Profiling consumers in the selected age group gives a deeper insight into the presented data. 

The main limitation of this study refers to somewhat confusing definition of pulses which could result in differences in understanding pulses as a food group. We suggest that in future studies as well as interventions this issue should be very carefully described and explained to consumers. Limitations may have arisen from the overall length and complexity of the survey questions that may have influenced the participants’ engagement. 

## 5. Conclusions

This study investigated the attitudes towards pulse consumption as well as the barriers and opportunities of introducing them or increasing their role in the daily nutrition of Polish young employees living in cities. Despite familiarity with traditional Polish dishes prepared from pulses, the image of this nutritionally and environmentally valuable food was influenced by the lack of habits of preparing them as well as perceived discomfort after eating. The majority of participants, especially women, were positive towards increasing pulse consumption and indicated many factors that could potentially impact that change. Women also proved to be more eager to substitute meat with pulses. This can suggest that consumers are open to adopt a more sustainable diet including a higher pulse intake; however, they need a nudge. Promotion and educational initiatives should focus on practical aspects such as recipes and cooking skills, especially culinary techniques which eliminate discomfort after eating. Satisfying nutritional needs cannot be seen only through the lens of preferences. Food choices must be more determined by the factors that create responsible and sustainable eating habits.

## Figures and Tables

**Table 1:** Socio-demographic and household characteristics of survey participants.

Characteristics	Total; *n* (%)	Characteristics	Total; *n* (%)
**Gender**		**Household’s financial situation**	
Female	534 (52)		
Male	493 (48)		
**Education**		Very good	58 (6)
Primary or vocational	40 (4)	Good	435 (42)
High school	272 (27)	Average	493 (48)
Bachelor’s degree	207 (20)	Rather bad	31 (3)
Master’s degree or higher	508 (49)	Very bad	10 (1)
**Size of place of residence (no. of inhabitants)**		**Type of employment**	
<100,000	496 (48)		
100,000–500,000	295 (29)	Full time	875 (85)
>500,000	236 (23)	Other	152 (15)
**Total household size (no. of persons)**		**Type of work**	
1	110 (11)		
2	274 (27)		
3	322 (31)	White collar	666 (65)
4	246 (24)	Blue collar	225 (22)
5 and more	75 (7)	Refusal	136 (13)
**Children under the age of 18**		**Diet**	
		No specific diet	784 (76)
		Limit animal products	163 (16)
		No meat	30 (3)
Yes	522 (51)	No animal products	10 (1)
No	505 (49)	Other	40 (4)

**Table 2:** Frequency of eating pulses and soy products in the total sample and 4 profiles.

	Total Sample	Profiles			
	N	F1	F2	F3	F4
	1027 (100%)	357 (35%)	313 (30%)	187 (18%)	170 (17%)
Beans	2.49	2.48	1.90	3.12	2.89
Peas	2.20	2.22	1.57	2.99	2.47
Lentils	2.04	1.91	1.15	3.04	2.85
Chickpeas	2.02	1.82	1.12	3.08	2.97
Tofu and soy products	1.68	1.44	1.09	3.05	1.78
Soy milk	1.67	1.47	1.08	3.16	1.56
**Average**	**2.02**	**1.89**	**1.32**	**3.07**	**2.42**

Assessment: 1.0–1.50—never; >1.50–2.50—several times a year; >2.50–3.50—several times a month; >3.50–4.0—several times a week. The average frequency for total sample and each profile are in bold.

**Table 3:** Respondents’ compliance with opinions and attitudes concerning pulse consumption.

Question	Agreement (%)		
Choose Your Opinion to the Following Statements	Definitely Not or Rather Not	Neutral	Definitely Yes or Rather Yes
In everyday cooking meat can be replaced by pulses	24	33	43
Pulses are suitable for daily meals	13	29	58
Nowadays times are different, there is no need to eat beans	39	38	23
Pulses (without meat) can be a base for a proper meal	12	24	64
Pulses are good only for people who do not eat meat	40	28	32
Eating pulses has become trendy	11	34	55
Dishes made of pulses are too common	45	35	20
Pulses are more environmentally friendly than meat	9	29	62
Substituting meat with pulses slows down climate change	20	41	39
Pulses are a good source of protein	6	22	72
Pulses are a cheaper source of nutrients than meat	10	34	56

## Appendix A

**Table A1:** Questionnaire structure.

Section	Question/Instruction	Description/Statements
Frequency (F)	“How often do you eat the following products in all forms means raw (dry), canned or in ready-to-eat dishes?” *Four-point scale: never/several times a year/several times a month/several times a week*	beans dry peas lentils chickpeas tofu and soy products soy milk
Attitudes (ATT)	(I) “Specify your opinion on the scale” *Scale without gradual: 1—unhealthy/distasteful and 5—healthy/tasty*	Pulses are healthy for most people Pulses are healthy for small children (aged 6–36 months) Pulses are tasty
	(II) “Choose your opinion on the following statements” *Five-level Likert-type scale: definitely not/rather not/neither yes nor not/rather yes/definitely yes*	In everyday cooking meat can be replaced by pulses Pulses are suitable for daily meals Times are different now, there is no need to eat beans now Pulses (without meat) can be a base of a proper meal Pulses are good only for people on a meatless diet Eating pulses has become a fashion Pulse dishes are too common Pulses are more environmentally friendly than meat Substituting meat with pulses slows down climate change Pulses are a good source of protein Pulses are a cheaper source of nutrients than meat
Barriers (BAR)	“What are the barriers of your pulse consumption?” *Possibility to select any number of answers*	I don’t like their taste I feel discomfort after eating themI don’t know how to prepare them These kind of dishes are not accepted at home I do not have the habit of eating them Long cooking time Other There are no barriers
Chances for potential growth (CH)	“Which of the following would make you eat more pulse-based dishes and reduce the proportion of meat and processed meat in your daily diet?” *Five-level Likert-type scale: definitely not/rather not/neither yes nor not/rather yes/definitely yes*	Gaining knowledge about the products’ nutritional value Gaining cooking skills Lack of discomfort after eating Getting to know new recipes A belief that I protect the environment that way Wider offer of RTE (Ready To Eat) foods Wider offer in restaurants Lower prices of products and dishes Educational campaign Producers advertisements

## References

[1] FAO (2016). Pulses: Nutritions Seeds for Sustainable Future.

[2] Górnicka M., Pierzynowska J., Wiśniewska M., Frąckiewicz J. (2011). An analysis of the consumption of dry leguminous vegetable seeds in Poland in 1999-2008 (in Polish with English summary). Bromatol. Chem. Toksykol..

[3] Thompson H.J. (2019). Dietary Bean Consumption and Human Health. Nutrients.

[4] Margier M., Georgé S., Hafnaoui N., Remond D., Nowicki M., Du Chaffaut L., Amiot M.J., Reboul E. (2018). Nutritional composition and bioactive content of legumes: Characterization of pulses frequently consumed in France and effect of the cooking method. Nutrients.

[5] Iqbal A., Khalil I.A., Ateeq N., Sayyar Khan M. (2006). Nutritional quality of important food legumes. Food Chem..

[6] Wallace T.C., Murray R., Zelman K.M. (2016). The nutritional value and health benefits of chickpeas and hummus. Nutrients.

[7] Curran J. (2012). The nutritional value and health benefits of pulses in relation to obesity, diabetes, heart disease and cancer. Br. J. Nutr..

[8] Boye J., Zare F., Pletch A. (2010). Pulse proteins: Processing, characterization, functional properties and applications in food and feed. Food Res. Int..

[9] Lynch H., Johnston C., Wharton C. (2018). Plant-based diets: Considerations for environmental impact, protein quality, and exercise performance. Nutrients.

[10] Ofuya Z.M., Akhidue Z. (2005). The role of Pulses in Human Nutrition. J. Appl. Sci. Environ. Manag..

[11] Ramdath D., Renwick S., Duncan A.M. (2016). The Role of Pulses in the Dietary Management of Diabetes. Can. J. Diabetes.

[12] Papandreou C., Becerra-Tomás N., Bulló M., Martínez-González M.Á., Corella D., Estruch R., Ros E., Arós F., Schroder H., Fitó M. (2019). Legume consumption and risk of all-cause, cardiovascular, and cancer mortality in the PREDIMED study. Clin. Nutr..

[13] Clark J.L., Taylor C.G., Zahradka P. (2018). Rebelling against the (insulin) resistance: A review of the proposed insulin-sensitizing actions of soybeans, chickpeas, and their bioactive compounds. Nutrients.

[14] Chen Y., Mcgee R., Vandemark G., Brick M., Thompson H.J. (2016). Dietary Fiber Analysis of Four Pulses Using AOAC 2011.25: Implications for Human Health. Nutrients.

[15] Flint H.J. (2012). The impact of nutrition on the human microbiome. Nutr. Rev..

[16] Rea K., Dinan T.G., Cryan J.F. (2016). The microbiome: A key regulator of stress and neuroinflammation. Neurobiol. Stress.

[17] Yadav B.S., Sharma A., Yadav R.B. (2010). Resistant starch content of conventionally boiled and pressure-cooked cereals, legumes and tubers. J. Food Sci. Technol..

[18] Kim S.J., De Souza R.J., Choo V.L., Ha V., Cozma A.I., Chiavaroli L., Mirrahimi A., Mejia S.B., Di Buono M., Bernstein A.M. (2016). Effects of dietary pulse consumption on body weight: A systematic review and meta-analysis of randomized controlled trials. Am. J. Clin. Nutr..

[19] Mollard R.C., Luhovyy B.L., Panahi S., Nunez M., Hanley A., Anderson G.H. (2012). Regular consumption of pulses for 8 weeks reduces metabolic syndrome risk factors in overweight and obese adults. Br. J. Nutr..

[20] Li S.S., Kendall C.W.C., De Souza R.J., Jayalath V.H., Cozma A.I., Ha V., Mirrahimi A., Chiavaroli L., Augustin L.S.A., Blanco Mejia S. (2014). Dietary pulses, satiety and food intake: A systematic review and meta-analysis of acute feeding trials. Obesity.

[21] McCrory M.A., Hamaker B.R., Lovejoy J.C., Eichelsdoerfer P.E. (2010). Pulse Consumption, Satiety and Weight Management. Am. Soc. Nutr..

[22] Mudryj A.N., Yu N., Aukema H.M. (2014). Nutritional and health benefits of pulses. Appl. Physiol. Nutr. Metab..

[23] Fabbri A.D.T., Crosby G.A. (2016). A review of the impact of preparation and cooking on the nutritional quality of vegetables and legumes. Int. J. Gastron. Food Sci..

[24] Garden-Robinson J., McNeal K. (2013). All about Beans.

[25] Polak R., Phillips E.M., Campbell A. (2015). Legumes: Health benefits and culinary approaches to increase intake. Clin. Diabetes.

[26] Gepts P., Bettinger R.L., Brush S.B., Damania A.B., Famula T.R., McGuire P.E., Qualset C.O. (2012). Introduction: The Domestication of Plants and Animals: Ten Unanswered Questions.

[27] Swinburn B.A., Kraak V.I., Allender S., Atkins V.J., Baker P.I., Bogard J.R., Brinsden H., Calvillo A., De Schutter O., Devarajan R. (2019). The Global Syndemic of Obesity, Undernutrition, and Climate Change: The Lancet Commission report. Lancet.

[28] Aleksandrowicz L., Green R., Joy E.J.M., Smith P., Haines A. (2016). The impacts of dietary change on greenhouse gas emissions, land use, water use, and health: A systematic review. PLoS ONE.

[29] McDermott J., Wyatt A.J. (2017). The role of pulses in sustainable and healthy food systems. Ann. N. Y. Acad. Sci..

[30] Reckling M., Hecker J., Bergkvist G., Watson C.A., Zander P., Schläfke N., Stoddard F.L., Eory V., Topp C.F.E., Maire J. (2015). A cropping system assessment framework—Evaluating effects of introducing legumes into crop rotations. Eur. J. Agron..

[31] Gummagolmath K.C., Sharma P., Patil S.M. (2013). Role of pulses in the food and nutritional security in India. J. Food Legum..

[32] FAO (2019). The State of the Wolrd’s Biodiversity for Food and Agriculture.

[33] Bahl P.N. (2015). Climate Change and Pulses: Approaches to Combat Its Impact. Agric. Res..

[34] Rizzo G. (2018). Soy, Soy Foods and Their Role in Vegetarian Diets. Nutrients.

[35] Willett W., Rockström J., Loken B., Springmann M., Lang T., Vermeulen S., Garnett T., Tilman D., DeClerck F., Wood A. (2019). Food in the Anthropocene: The EAT–Lancet Commission on Healthy Diets from Sustainable Food Systems. Lancet.

[36] Rejman K., Kaczorowska J., Halicka E., Laskowski W. (2019). Do Europeans consider sustainability when making food choices? A survey of Polish city-dwellers. Public Health Nutr..

[37] Halicka E., Kaczorowska J., Szczebyło A. (2019). Sustainable Food Consumption in Rural Households with Children (in Polish with English summary). Village Agric..

[38] IPSOS REID (2010). Factors Influencing Pulse Consumption in Canada.

[39] Jallinoja P., Niva M., Latvala T. (2016). Future of sustainable eating? Examining the potential for expanding bean eating in a meat-eating culture. Futures.

[40] Fiore M. (2017). Legumes Consumption among Young and Adult Residents in Sicily (South Italy): Evidence and Predictive Factors. J. Nutr. Health Food Sci..

[41] Vainio A., Niva M., Jallinoja P., Latvala T. (2016). From beef to beans: Eating motives and the replacement of animal proteins with plant proteins among Finnish consumers. Appetite.

[42] Melendrez-ruiz J., Buatois Q., Chambaron S., Monnery-patris S. (2019). French consumers know the benefits of pulses, but do not choose them: An exploratory study combining indirect and direct approaches. Appetite.

[43] Marinangeli C.P.F., Curran J., Barr S.I., Slavin J., Puri S., Swaminathan S., Tapsell L., Patterson C.A. (2017). Enhancing nutrition with pulses: Defining a recommended serving size for adults. Nutr. Rev..

[44] Garnett T. (2014). What Is a Sustainable Healthy Diet? A Discussion Paper.

[45] FAO, WHO (2019). Sustainable Healthy Diets.

[46] Kramer G., Durlinger B., Kuling L., van Zeist W., Blonk H., Broekema R., Halevy S. (2017). Eating for 2 Degrees New and Updated Livewell Plates.

[47] Derbyshire E.J. (2017). Flexitarian Diets and Health: A Review of the evidence-Based Literature. Front. Nutr..

[48] Aiking H., de Boer J. (2018). The next protein transition. Trends Food Sci. Technol..

[49] Allès B., Baudry J., Méjean C., Touvier M., Péneau S., Hercberg S., Kesse-Guyot E. (2017). Comparison of sociodemographic and nutritional characteristics between self-reported vegetarians, vegans, and meat-eaters from the nutrinet-santé study. Nutrients.

[50] (2019). Roślinnie-Jemy Raport—Postawy Konsumentów Wobec Produktów i dań Roślinnych.

[51] Ipsos MORI (2018). What Does it Mean to Consumers? An Exploration into Diets around the World.

[52] Weibel C., Ohnmacht T., Schaffner D., Kossmann K. (2019). Reducing individual meat consumption: An integrated phase model approach. Food Qual. Prefer..

[53] de Boer J., Aiking H. (2018). Prospects for pro-environmental protein consumption in Europe: Cultural, culinary, economic and psychological factors. Appetite.

[54] Halicka E., Rejman K. (2010). Food consumption patterns in Poland (in Polish with English summary). Village Agric..

[55] Mills S., White M., Brown H., Wrieden W., Kwasnicka D., Halligan J., Robalino S., Adams J. (2017). Health and social determinants and outcomes of home cooking: A systematic review of observational studies. Appetite.

[56] Figueira N., Curtain F., Beck E., Grafenauer S. (2019). Consumer Understanding and Culinary Use of Legumes in Australia. Nutrients.

[57] Winham D.M., Tisue M.E., Palmer S.M., Cichy K.A., Shelley M.C. (2019). Dry bean preferences and attitudes among midwest hispanic and non-hispanic white women. Nutrients.

[58] Winham D.M., Hutchins A.M., Dougherty M.K. (2018). Arizona Registered Dietitians Show Gaps in Knowledge of Bean Health Benefits. Nutrients.

[59] Lea E., Worsley A., Crawford D. (2005). Australian adult consumers’ beliefs about plant foods: A qualitative study. Health Educ. Behav..

[60] Winham D.M., Hutchins A.M. (2011). Perceptions of flatulence from bean consumption among adults in 3 feeding studies. Nutr. J..

[61] IŻŻ Pyramid of Healthy Eating and Physical Activity. http://www.izz.waw.pl/zasady-prawidowego-ywienia.

[62] USDA My Plate. https://www.choosemyplate.gov/eathealthy/WhatIsMyPlate.

[63] Rose D., Heller M.C., Roberto C.A. (2019). Position of the Society for Nutrition Education and Behavior: The Importance of Including Environmental Sustainability in Dietary Guidance. J. Nutr. Educ. Behav..

[64] Havemeier S., Erickson J., Slavin J. (2017). Dietary guidance for pulses: The challenge and opportunity to be part of both the vegetable and protein food groups. Ann. N. Y. Acad. Sci..

